# Focal therapy for prostate cancer: revolution or evolution?

**DOI:** 10.1186/1471-2490-9-2

**Published:** 2009-04-23

**Authors:** Ryan Turpen, Charles J Rosser

**Affiliations:** 1 Department of Urology, University of Florida, Gainesville, Florida, USA

## Abstract

The face of prostate cancer has been dramatically changed since the late 1980s when PSA was introduced as a clinical screening tool. More men are diagnosed with small foci of cancers instead of the advanced disease evident prior to PSA screening. Treatment options for these smaller tumors consist of expectant management, radiation therapy (brachytherapy and external beam radiotherapy) and surgery (cryosurgical ablation and radical prostatectomy). In the highly select patient, cancer specific survival employing any of these treatment options is excellent, however morbidity from these interventions are significant. Thus, the idea of treating only the cancer within the prostate and sparing the non-cancerous tissue in the prostate is quite appealing, yet controversial. Moving forward if we are to embrace the focal treatment of prostate cancer we must: be able to accurately identify index lesions within the prostate, image cancers within the prostate and methodically study the litany of focal therapeutic options available.

## Background

As defined by the International Task Force on Prostate Cancer and the Focal Lesion Paradigm, the goal of focal therapy for prostate cancer would be to "selectively ablate(s) known disease and preserve(s) existing functions, with the overall objective of minimizing lifetime morbidity without compromising life expectancy" [1]. Several, new treatment modalities for localized prostate cancer with encouraging efficacy and reduced morbidity compared to conventional treatment options may have the potential of accomplishing this goal [2].

Focal therapy for the treatment of prostate cancer has been called the 'male lumpectomy', an analogue to women's breast lumpectomy for the treatment of breast cancer [3]. In a lumpectomy, the breast tumor and some of the normal tissue that surrounds it are resected, thus sparing significant portion of the breast. This breast conserving surgery is associated with less morbidity. However, most women opting for breast lumpectomy receive 5 to 7 weeks of local radiation therapy shortly after lumpectomy in order to eliminate any cancer cells that may linger in the conserved breast. Two large prospective randomized control trials showed that women with small breast cancers (under 4 centimeters) treated with lumpectomy plus radiation therapy were just as likely to be alive and disease-free 20 years later as women who had had mastectomies. Additional, neoadjuvant chemotherapy may be part of the treatment regimen when breast lumpectomy is performed [4,5]. To date, most discussions on 'male lumpectomy' are devoid of radiation therapy or chemotherapy. With that said, is focal therapy for prostate cancer really similar to breast lumpectomy? Furthermore, is the talk of focal therapy for prostate cancer, heresy? Herein, we discuss critical pathologic and radiologic findings conducive to focal therapy and we briefly discuss the results of a variety of therapeutic options utilized for focal therapy.

## Discussion

Successful adoption of focal therapy for the treatment of prostate cancer will hinge on several critical issues: 1) can we accurately identify index lesions within the prostate, 2) can we reliably image cancers within the prostate, 3) long-term efficacy of the technology to eradicate cancer, 4) appropriate follow-up of patients treated with focal therapy, and 5) limitations of PSA following therapy and how to detect recurrent/persistent disease.

### Pathology

Extensive, bilateral disease precludes focal therapy. Multifocal prostate cancer has been reported in 67% to 87% of all cases of contemporary radical retropubic prostatectomy [6]. Thus, the ideal candidate considered for focal therapy should have unilateral disease. A recent study out of Duke University reported that in 1,386 prostatectomy specimens, 18% of the tumors were unilateral only. On multivariate analysis, race, pathologic Gleason score, surgical margin status and percentage of tumor involvement as independent predictors of unilateral disease [7]. It is noted that pathologic Gleason score and surgical margins are not known when selecting patients for focal therapy.

In order to implement focal therapies, cancer must be accurately and reliably mapped within the gland. Initially, transperineal 3D mapping biopsies were reported to effectively exclude patients with clinically significant unsuspected cancer outside the area destined to be ablated more so than repeat transrectal ultrasound (TRUS) guided biopsies [8]. Though not corroborated by whole-mount prostatectomy specimens, Onik reported the use of transperineal 3D mapping biopsy used as an additional staging procedure prior to focal prostate cancer therapy. One hundred ten patients, all of whom had unilateral disease on TRUS biopsies, were restaged using the 3D mapping method prior to focal therapy. Bilateral cancer was demonstrated in 60 patients (55%, all of whom had only unilateral cancer shown on TRUS biopsy). The Gleason score was increased in 25 patients (23%) over the Gleason score found on TRUS biopsy [9], thus suggesting transperineal prostate mapping provides better pathologic assessment of the entire prostate gland.

Perhaps the 3D nature in which the biopsies are obtained not the route the biopsies are obtained (i.e., transrectal versus transperineal) is not the critical factor. Researchers using the TargetScan 3D transrectal ultrasound and prostatic biopsy system correctly characterize 88% of ex vivo prostatic octants in regards to whether or not they contained cancer [10]. These 3-D mapping biopsies have been suggested in some of the focal therapy clinical trials discussed at the 1^st ^International Workshop on Focal Therapy and Imaging of Prostate Cancer.

Though standard imaging with ultrasound have limited sensitivity of identifying pathologic proven tumor within the prostate gland, novel techniques are being investigated with prostate biopsying, including the use of ultrasonographic contrast agents, color Doppler, power Doppler, and MR spectroscopy image-directed TRUS-guided biopsy in hopes of better identifying cancer at the time of prostate biopsy [11,12].

### Imaging

Equally important to the success of focal therapy of localized prostate cancer is imaging and its ability to more accurately provide information regarding tumor presence and localization before and after treatment. Tumor localization using imaging modalities allows for treatment of the area(s) involved with cancer and sparing of the normal, non-cancerous tissue. Our current radiologic staging modalities (i.e., prostate ultrasound and computed tomography of the pelvis) lack sensitivity in depicting the presence and location of localized disease [13].

Previous studies reported a variable sensitivity of magnetic resonance imaging (MRI) in localizing disease within the prostate [14,15]. Recently, the introduction of functional imaging techniques such as dynamic contrast-enhanced (DCE) MRI, diffusion-weighted imaging (DWI) and magnetic resonance spectroscopic imaging (MRSI) have been studied to improve the detection and localization of prostate cancer. Specifically, DCE MRI was assessed in 53 patients with suspected prostate cancer. The wash-in rate value was greater in cancer tissue (9.2/second) than in three normal tissues (3.3/second, 6.7/second, and 3.2/second, respectively; P < 0.001). The sensitivity and specificity of detecting prostate cancer were greater on parametric imaging of the wash-in rate compared to T2-weighted imaging in the entire prostate (96% and 82% vs. 65% and 60%, respectively) [16]. As for DWI MRI, T2-weighted imaging and DWI were performed in 49 patients before radical prostatectomy using an endorectal coil at 1.5 T in a prospective trial. Sensitivity for prostate cancer detection was significantly higher with T2 plus DWI (81%) than with T2 imaging alone (54%), with T2 plus DWI showing only a slight loss in specificity compared with T2 imaging alone (84% vs. 91%, respectively) [17].

MRI, MRSI and DCE-MRI with a 3-Tesla whole-body scanner were performed in 30 patients with biopsy-proven prostate cancer before radical prostatectomy. The high signal-to-noise ratio (SNR) at 3 Tesla provided T2-weighted turbo spin echo (TSE) images with excellent anatomical detail (in-plane voxel size of 0.22 × 0.22 mm) and good T2 contrast, whereas dynamic contrast-enhanced images showed good temporal resolution [18]. The combination of vascular information from DCE-MRI or DWI MRI and metabolic data from MRSI has excellent potential for improved accuracy in delineating and staging prostate carcinoma.

### Cryotherapy

Though fascinating, delving into the history and biology of cryotherapy is not part of this review. Recently, the AUA released its best practice statement on cryosurgery for the treatment of localized prostate cancer [19]. A cornerstone of this report was the large, retrospective series reported by Cohen with a median follow-up of greater than 12 years. In this report, 370 patients with T1 to T3 prostate cancer treated consecutively from 1991 to 1996 with cryosurgery as primary monotherapy was analyzed. Kaplan-Meier analysis demonstrated a biochemical disease-free survival rate at 10 years of 80.56%, 74.16%, and 45.54% for low, moderate, and high-risk groups, respectively. The 10-year negative biopsy rate was 76.96% [20]. Another large, retrospective series was reported by Bahn and colleagues. In this report, the charts of 590 consecutive patients who underwent primary cryosurgical ablation for definitive management were reviewed. The mean follow-up of the cohort was 5.4 years. The percentages of patients with low-, medium-, and high-risk prostate cancer were 15.9%, 30.3%, and 53.7%, respectively. Using a PSA-based definition of biochemical failure of 0.5 ng/mL, results were as follows: the 7-year actuarial bDFS for low-, medium-, and high-risk patients were 61%, 68%, and 61%, respectively. The rate of positive biopsy was 13%. Hormonal therapy was given to 91.5% of the subjects before treatment to downsize the gland and consisted of luteinizing hormone-releasing hormone, combined with an antiandrogen agent 3 months to 1 year before cryoablation. Though hormonal therapy was not continued on any patient after cryoablation, it is still difficult to interpret early serum PSA results in this cohort. Lastly it was noted that 4.3% of subjects reported post-operative incontinence, 94.9%of subjects reported post-operative erectile dysfunction and < 0.1% of subjects developed a post-operative fistula. Thus, the rates of morbidity were modest, and no serious complications were observed [21]. These encouraging results with whole gland treatment have sparked an interest in utilizing this treatment modality for the focal treatment of prostate cancer.

Focal cryoablation was planned to encompass the area of known tumor based on staging biopsies. Forty-eight patients with at least 2-year follow-up had focal cryoablation. Mean follow-up was 4.5 years. Ninety-four percent of the patients treated had stable PSAs according to American Society of Therapeutic Radiology and Oncology (ASTRO) criteria. Of the 24 patients with stable PSAs who were routinely biopsied (n = 24) all were negative. No local recurrences were noted in areas treated. Potency was maintained to the satisfaction of the patient in of 36 of 40 patients who were potent preoperatively. Of the 48, all were continent [22]. It is the limited morbidity that makes this treatment option quite attractive. These encouraging results have prompted the initiation of two large, multicenter trials assessing the feasibility and efficacy of performing focal cryotherapy in subjects with localized, low-risk prostate cancer.

The use of hormone therapy must be accounted for when interpreting the results from the above studies. In the study by Onik, patients on combined hormone therapy had therapy stopped immediately after treatment in all cases. Nonetheless, the 3-month data should be cautiously interpreted due to potential residual effects of neoadjuvant hormone therapy [22]. A 2005 study evaluating men with high-risk features for prostate carcinoma, the majority of study participants (67.7%) received neoadjuvant hormones. Despite this, no significant difference was seen in biochemical recurrence-free survival between those who received hormones and those who did not [23]. Regardless, use of hormones must be taken into consideration when interpreting PSA-recurrence or biopsy results in these patients.

### High-intensity focused ultrasound

First introduced in the 1940s, high-intensity focused ultrasound (HiFU) uses energy focused to a specific point within the prostate, thus enabling targeted destruction of prostatic tissue. Currently, two HiFU devices are attempting to get FDA approval for the treatment of prostate cancer in the US. However, HiFU therapy for localized prostate cancer has been widely accepted in Europe and Asia. In a large Phase II/III prospective multicenter study, Thuroff reported the results of 402 patients with stage T1-2, Nx-0, M0 prostate cancer treated with HiFU. Follow-up was extremely short at approximately 1 year, but the negative biopsy rate observed in the T1-2 primary-care population was 87.2% [24]. The HiFU series with the longest follow-up comes out of the University of Regensburg in Germany. In their report, Blana reports on the outcomes of 163 patients with clinical Stage T1-T2N0M0, biopsy-proven, localized prostate cancer, with a serum prostate-specific antigen (PSA) level of ≤ 20 ng/mL, Gleason score of ≤ 7, treated with HiFU. Median follow-up was 4.8 years. Of the 163 patients, 86.4% achieved a PSA nadir of < 1 ng/mL and 92.7% had negative post-treatment biopsy findings. The biochemical recurrence free survival rate at 5 years was 75%, with salvage treatment initiated for 12% of the patients. On multivariate analysis, the pretreatment PSA level was the only statistically significant predictive factor of recurrence (P = 0.005) [25].

Similar to focal cryoablation, focal HiFU is planned to encompass the area of known tumor based on staging biopsies. In 2008, Muto and others published a study on 70 patients treated with HiFU. Twenty-nine of these patients had unilateral disease as determined by multi-regional biopsies. These patients received focal therapy while the remaining patients received whole organ ablation. Scheduled biopsies were performed at 6 and 12 months following treatment. After 12 months 84% of patients who received whole therapy were biopsy negative versus 77% in the focal therapy group. Using the ASTRO definition, the 2-year biochemical recurrence free survival rates for patients at low and intermediate risk were 91% and 50% for whole therapy, respectively. For the focal therapy group, biochemical recurrence free survival rates were 83% and 54%, respectively at 2 years. The authors also noted in this study that whole, but not focal therapy, resulted in a continuous decline in serum testosterone levels. Of the 52 patients who were continent prior to HiFU, 49 (94%) subjects were continent after HiFU therapy. Morbidity of whole gland HiFU compared to focal gland HiFU was not significantly different [26]. These results are promising and support focal cryotherapy as a therapeutic modality in subjects with localized, low-risk prostate cancer.

Similar with cryosurgery, in patients who received hormone therapy, caution must be taken when interpreting these results. The study by Thuroff excluded patients who had previous orchiectomy or hormone therapy [24]. Muto does not account for hormone therapy in his results despite reporting 24 of 70 patients with hormone therapy at time of treatment [26].

### Vascular Targeted Photodynamic Therapy

Vascular targeted photodynamic therapy (VTP) involves the generation of cytotoxic agents in situ that results in cell death and tissue ablation. This is achieved by systemic or local administration of a photosensitizing drug that is activated with light of a specific wavelength. The photosensitizer Tookad^® ^(WST09) has been studied in the VTP of prostate cancer. Huang and colleagues demonstrated that Tookad-VTP could destroy a clinically-significant volume of normal canine prostate tissue [27]. In a phase I clinical trial conducted in Europe and Canada, Trachtenberg and others report the safety and efficacy of VTP in 28 patients with histological proven recurrent prostate cancer after definitive radiotherapy. In the study, response to VTP was assessed using contrast-enhanced MRI and transrectal ultrasound guided biopsies targeting areas of lesion formation in addition to monitoring serum PSA levels. Areas of necrosis were documented on MRI scans. Serum PSA levels decreased in the complete responders, however, PSA levels in partial and nonresponders remained similar to baseline levels, regardless of light doses received. Side-effects of the TOOKAD-VTP treatment were modest, generally self-limited, and compared favorably with other salvage methods. However, two patients did develop a rectourethral fistula, which translates into a remarkably high fistula rate. The authors speculate that this high fistula rate may be explained by an EBRT induced prostatitis, resulting in higher drug concentration in blood pooling within the rectum [28]. Regardless, efforts must be aimed at decreasing the morbidity reported in this most recent study.

Recently, Emberton presented results of a phase I trial using VTP to treat low volume, primary prostate cancer. Preliminary results are encouraging, however the authors readily admit refinements in the technique are needed prior to embarking on a larger clinical trial [29]. The encouraging finding in this study was that Tookad-VTP induced lesions were able to be accurately shaped using a single light source, thus highlighting its potential role in focal prostate therapy.

## Summary

The face of prostate cancer has been dramatically changed since the late 1980s when PSA was introduced as a clinical screening tool. More men are diagnosed with localized prostate cancer [30] and smaller volume, non-aggressive prostate cancers [31]. It is unlikely that these cancers will adversely affect the individuals overall survival, thus expectant management or now focal therapy may be an excellent treatment modality for this very select cohort. A key remains the ability to identify candidates for focal therapy. The International Task Force on Prostate Cancer and the Focal Lesion Paradigm has proposed a clinical definition of suitable candidates for focal therapy (Table 1) [32]. But for those individuals opting for definitive therapy, perhaps focal therapy utilizing any of the modalities discussed above may be an option [33]. It is noted that advancements in the delivery of external beam radiation and brachytherapy are enabling these therapies to be considered in the focal therapy armamentarium.

**Table 1:** Ideal Candidates for Focal Therapy*

Serum PSA	PSA < 10 ng/mL, PSAD < 0.15 ng/mL/g
Clinical Stage	T1NxMx or T2aNxMx

Pathologic evaluation/Gleason score^	3+3 or less (no grade 4 or 5)

	No more than 2 adjacent regions positive for cancer

	Total length of cancer < 10 mm total and < 7 mm in any 1 core; < 1/3 of cores positive for cancer

Radiologic Imaging (MRI +/- MRSI)	Largest dimension < 15 mm if prostate volume > 25 grams or < 10 mm if volume < 25 grams. Capsular contact < 5 mm on axial imaging. No signs of extracapsular extension or seminal vesicle invasion

* adapted from Sartor, 2008.

^10 core minimum biopsy schema, plus 2 additional cores for every 10 grams of prostate > 40 grams (max of 18 cores)

Though focal therapy of the prostate only treats a portion of the prostate it has been referred to as the 'male lumpectomy', but one must remember women who undergo lumpectomy usually will have other local or systemic therapies to ensure eradication of disease. Since men who are undergoing 'male lumpectomy' rarely undergo these addition therapies, comparison with women undergoing a lumpectomy should be limited. However, through rigorous clinical scrutiny the ideal treatment combinations were identified for breast cancer that successfully treated the cancer and limited the morbidity associated with more radical therapies. A strong push must be made in the Urologic community to rigorously scrutinize focal therapy in men with prostate cancer.

As previously stated, successfully adoption of focal therapy for the treatment of prostate cancer will hinge on two critical issues: 1) accurately identifying index lesions within the prostate, 2) reliably imaging cancers within the prostate, 3) long-term efficacy of the technology to eradicate cancer, 4) appropriate follow-up of patients treated with focal therapy, 5) limitations of PSA following therapy and how to detect recurrent/persistent disease. Solving these critical issues not only will assist with pretreatment counseling of these patients, but could be used as effective monitors for ideal end points in cancer therapy. In addition, optimal patient selection using pretreatment risk stratification must be developed to ensure the treatment of subjects who would gain the most out of focal therapy. Despite our limitations in identifying disease that can be treated by focal therapy, several clinical trials utilizing some of the promising therapeutic modalities mentioned above are on the horizon. Hopefully with careful monitoring and critical review of these trials we can better determine if focal therapy is an appropriate treatment option to offer patients with localized, low-grade prostate cancer.

## Abbreviations

PSA: prostate specific antigen; 3D: 3 dimension; TRUS: transrectal ultrasound; MRI: magnetic resonance imaging; DCE: dynamic contrast-enhanced; MRSI: magnetic resonance spectroscopic imaging; DWI: diffusion-weighted imaging; SNR: signal-to-noise ratio; AUA: American Urological Association; ASTRO: American Society of Therapeutic Radiology and Oncology; HiFU: high-intensity focused ultrasound; FDA: Food and Drug Administration; DFS: disease free survival; VTP: Vascular targeted photodynamic therapy; TSE: turbo spin echo.

## Competing interests

The authors declare that they have no competing interests.

## Authors' contributions

RT gathered all data and wrote the manuscript. CJR developed and was responsible for overseeing the project.

## Pre-publication history

The pre-publication history for this paper can be accessed here:


